# Recent developments and future directions in meta-analysis of differential gene expression in livestock RNA-Seq

**DOI:** 10.3389/fgene.2022.983043

**Published:** 2022-09-19

**Authors:** Brittney N. Keel, Amanda K. Lindholm-Perry

**Affiliations:** USDA-ARS, Roman L. Hruska U.S. Meat Animal Research Center, Clay Center, NE, United States

**Keywords:** RNA-seq, meta-analysis, livestock, *p*-value combination, gene expression

## Abstract

Decreases in the costs of high-throughput sequencing technologies have led to continually increasing numbers of livestock RNA-Seq studies in the last decade. Although the number of studies has increased dramatically, most livestock RNA-Seq experiments are limited by cost to a small number of biological replicates. Meta-analysis procedures can be used to integrate and jointly analyze data from multiple independent studies. Meta-analyses increase the sample size, which in turn increase both statistical power and robustness of the results. In this work, we discuss cutting edge approaches to combining results from multiple independent RNA-Seq studies to improve livestock transcriptomics research. We review currently published RNA-Seq meta-analyses in livestock, describe many of the key issues specific to RNA-Seq meta-analysis in livestock species, and discuss future perspectives.

## 1 Introduction

**FIGURE 1:**
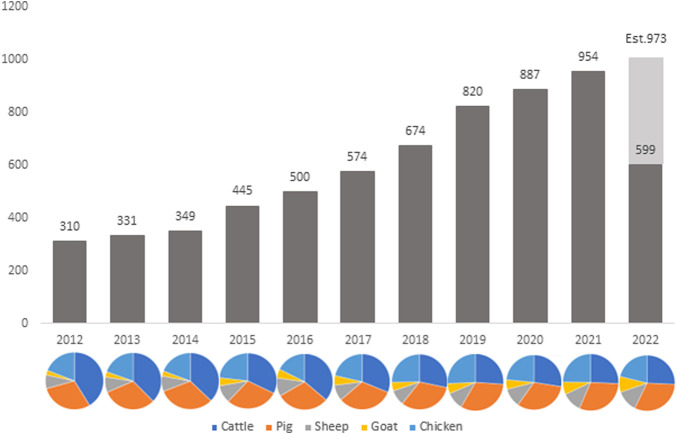
Number of published livestock transcriptome studies by year since 2012. Data was compiled via PubMed search: “TRANSCRIPTOME” and “species” and “year” (accessed 12 August 2022).

**FIGURE 2:**
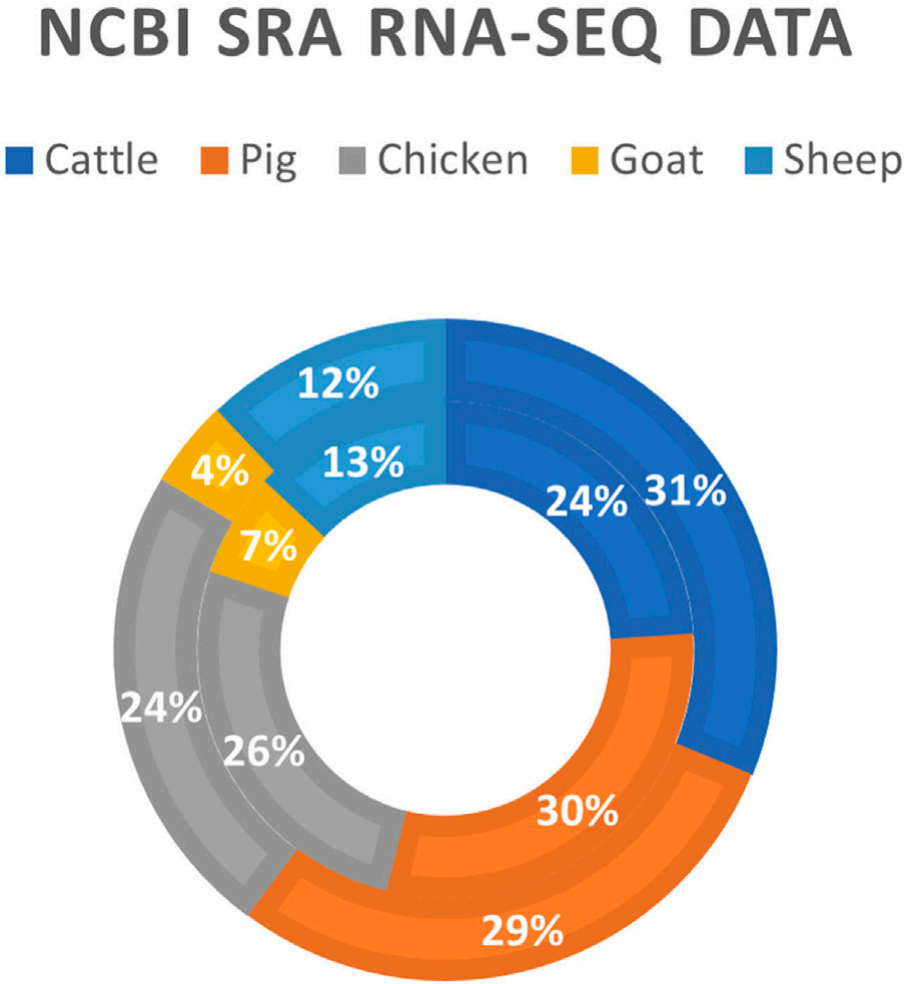
Livestock RNA-Seq datasets in NCBI SRA database. Data was generated using the online “Run Selector” tool on the NCBI SRA website (https://www.ncbi.nlm.nih.gov/sra; accessed 12 August 2022). The outer ring represents the number of biosamples in the database (*N* = 59,634), and the inner ring represents the number of distinct bioprojects in the database (*N* = 3,130).

The development of RNA-sequencing (RNA-Seq) as a high-throughput gene expression quantification technology has been crucial in the advancement of livestock genomics research. In the last decade, the number of annually published livestock transcriptome studies has nearly tripled (Figure 1). For most of these studies, raw sequence data is stored in public repositories, such as National Center for Biotechnology Information Sequence Read Archive (NCBI SRA). To-date, NCBI SRA houses RNA-Seq data from over 3,100 different livestock projects, comprised of approximately 59,000 samples (Figure 2). In addition, multiple consortiums, including Functional Annotation of Animal Genomes (FAANG; Giuffra et al., 2019) and Farm Animal Genotype-Tissue Expression (FarmGTEx; Liu et al., 2021), are continuing to collect, curate, and house RNA-Seq data for multiple livestock species. Data from these resources is easily accessible and can be utilized for integrated analyses to generate new knowledge and scientific findings.

Despite its widespread use, challenges remain in RNA-Seq data analysis. One major issue that has been observed in gene expression studies, in livestock and other species, is non-reproducibility of results (Shi et al., 2008). Due to sequencing costs, livestock RNA-Seq experiments are typically performed on a small number of biological replicates, which limits their power to detect differentially expressed genes (DEG) between experimental conditions. In addition to small numbers of replicates, inter-study variability due to technical differences (e.g., sample preparation, library protocols, batch effects) as well as biological differences (e.g., environmental, management, and genetic effects) also contributes to reproducibility issues.

One way to improve reproducibility of RNA-Seq is by integrating data from multiple independent studies via meta-analysis. It should be noted that the term meta-analysis is an all-encompassing term, used to describe a systematic synthesis of quantitative results from different empirical studies. Hence, meta-analysis can be used to describe a large breadth of analyses with different goals. In this review, we focus specifically on meta-analysis procedures for integrating RNA-Seq data across independent studies to identify differential gene expression.

Meta-analysis procedures increase the sample size by incorporating samples from different studies, increasing both statistical power and robustness of the results. Recently, the number of publications involving differential gene expression meta-analysis has increased drastically, with most studies conducted on human samples (Toro-Domínguez et al., 2021). In humans, meta-analysis has been widely used in identification of biomarkers related cancer (Chen et al., 2014; Bell et al., 2017; Kori and Arga, 2018) and other diseases, including hypertension (Huan et al., 2015), Alzheimer’s disease (Su et al., 2019; Wan et al., 2020), autoimmune disorders (Kröger et al., 2016), and schizophrenia (Piras et al., 2019).

To-date, few livestock RNA-Seq studies have utilized meta-analysis procedures to analyze differential gene expression. This is likely due to several issues, including, but not limited to, high levels of inter-study variation, availability of metadata, and limited technical guidance for conducting a meta-analysis. In this review, we discuss many of the key issues specific to RNA-Seq meta-analysis in livestock species. The first two issues are related to data acquisition and pre-processing, while the remaining issues relate to choosing a meta-analysis procedure and interpretation of results. Lastly, we discuss other points related to meta-analysis of livestock RNA-Seq data, including additional applications of meta-analysis procedures outside of differential expression analysis, new methodologies for large scale RNA-Seq, and single-cell RNA-Seq.

## 2 Applications of RNA-Seq differential gene expression meta-analysis in livestock

As described by Toro-Domínguez et al. (2021), a wide range of contexts exist for meta-analysis of gene expression data. In this work, we will focus on the use of meta-analysis of RNA-Seq data across multiple studies with the same phenotypic groups in order to increase the statistical power to detect genes showing consistent differences between groups. To-date, four livestock studies have reported the use of RNA-Seq meta-analysis procedures for differential expression analysis, all of which are cattle studies (Keel et al., 2018; Lindholm-Perry et al., 2020; Ghahramani et al., 2021; Lindholm-Perry et al., 2022). Traits of interest in these studies include those related to feed efficiency and lactation.

There have been numerous transcriptome studies that aim to identify DEG related to feed efficiency in beef cattle. Minimal overlap in the results from these studies is an ongoing issue. In order to identify DEG predictions that would be more robust across the beef cattle industry, companion studies in skeletal muscle (Keel et al., 2018) and mesenteric fat (Lindholm-Perry et al., 2020) identified DEG associated with body weight gain and feed intake in beef steers across five different cohorts of crossbred animals reared at the same facility. Data used in these studies included nineteen beef breeds from the GPE population (Retallick et al., 2017), as well as both fall and spring seasons over 3 years. Several of the DEG in both skeletal muscle and mesenteric fat had been previously identified as candidate genes for feed efficiency or DEG associated with feed efficiency in livestock.

In addition to the aforementioned studies, where data from multiple cohorts reared in the same facility were utilized, meta-analysis techniques have been used to combine data across independent studies with common aim to detect DEG associated with cattle feed efficiency. Differentially expressed genes in the rumen epithelium of beef steers with high and low residual feed intake (RFI) phenotypes (Lindholm-Perry et al., 2022) were identified from a meta-analysis of two unrelated and physically distant populations, one located in the U.S. and the other in Canada. A total of 83 DEG were identified in the meta-analysis, compared to 12 and 119 DEG in the individual U.S. and Canadian studies, respectively. Twenty of the DEG from the meta-analysis were classified as robust, meaning they passed a jackknife sensitivity test (for details on jackknife sensitivity testing see Section 4.2). These robust DEG were not identified in either of the individual analyses. Several DEG from the meta-analysis were involved in TORC2 signaling and proteasomal ubiquitin-independent protein catabolic biological processes. While gene ontology and pathway analyses of DEG in the individual studies did not identify these mechanisms, protein turnover via mTOR and ubiquitin-proteosome pathways have been identified as mechanisms involved in RFI in the rumen tissue of beef cattle in another study (Elolimy et al., 2019). This suggests that the meta-analysis approach can facilitate the discovery of more robust DEG through increased statistical power.

Transcriptomic studies related to bovine lactation have also taken advantage of meta-analysis approaches. Ghahramani et al. (2021) combined meta-analysis data from both publicly available RNA-Seq and microarray to investigate DEG associated with *E. coli* mastitis in dairy cattle. Separate meta-analyses were conducted for RNA-Seq (2 datasets) and microarray (6 datasets). A total of 360 DEG were common between the two meta-analyses. Common DEG were subjected to multiple downstream analyses, including ontology, protein-protein interaction (PPI) network analysis, and co-expression network analysis. Many of the significant biological pathways and DEG that were hubs in the PPI networks had been previously associated with mastitis in the literature, but no single study was able to identify all of them at once, indicating the meta-analysis provides a more robust biosignature.

## 3 Key issues in RNA-Seq meta-analysis workflow

In this section we discuss key steps and important considerations in the RNA-Seq meta-analysis workflow.

### 3.1 Identifying data sets and data acquisition

Eligibility criteria for inclusion of data sets is dependent on the aim of the study. Typical biological criteria include species, breed, disease status, treatment, tissue, age, and/or phenotypic group. Public repositories, such as the NCBI SRA database, have search options to easily identify data sets linked to specific keywords and ontologies. Careful attention should be given to the metadata associated with each data set and how different parameters could affect the results of the study. For example, if the goal of a given meta-analysis is to investigate differential gene expression associated with feed efficiency in a specific tissue, using a data set where animals have been inoculated with a bacteria or virus may not be appropriate due to feeding behavior differences associated with the disease state of the animal. In addition, diet (i.e., forage versus concentrate diets for cattle) and sampling location within a large tissue like the liver may also significantly affect gene expression.

One major issue with data in publicly available omics repositories, which is not unique to livestock, is the incompleteness of dataset descriptions, called metadata. Metadata can include many different pieces of information, including sources of data, dates of data collection, methods used, etc., (Koltes et al., 2019). Although stakeholders have come together to design and jointly endorse a concise and measurable set of guidelines for metadata, referred to as FAIR Data Principles (Wilkinson et al., 2016), the degree to which the research community follows these principles is varied. Recently, Rajesh et al. (2021) evaluated the completeness of public metadata accompanying transcriptomic data of patients with sepsis and corresponding controls (3,125 samples across 29 data sets). They found that, on average, only 65% of clinical phenotypes were reported in the publication and/or public repository, with 35% of the information thus being lost from the publication to the repository.

Metadata standards and infrastructure are crucial for meta-analysis across data sets. Under the Open Science initiative (Nosek et al., 2015), funding agencies and journals have begun to require that data used in a study be made publicly available, but in most databases the number of metadata entries required is still minimal. In order to incentivize authors to submit high-quality metadata, a prototype streamlined workflow for conversion of European Nucleotide Archive (ENA) genomic metadata into a data manuscript has been proposed (Dimitrova et al., 2021). Currently, this workflow is focused on genomic data, but future plans are to expand the workflow to include other repositories and data types.

To support submission of standardized rich metadata in animal genomics, the Functional Annotation of Animal Genomes (FAANG) consortium has developed the FAANG Data Portal (Harrison et al., 2021). The Data Portal offers open access to a wealth of data following FAIR Data Principles produced by an ever-growing number of FAANG consortia. To ensure that data submissions are of high-quality with complete metadata, the portal includes a contextual metadata validation. Changes to the metadata standards in the Data Portal can be proposed by anyone in the research community via their GitHub page. Adoption of metadata practices like those of ENA and FAANG, described herein, should be considered by other public repositories to facilitate the reuse of omics datasets.

### 3.2 Data preprocessing

Data preprocessing is an important step in the meta-analysis process. Unfortunately, there is no optimal pipeline for the variety of different applications and analysis scenarios in which RNA-Seq can be used, and preprocessing protocols may vary greatly from study to study (Conesa et al., 2016). For meta-analysis, data preprocessing should be as standardized as possible between studies in order to minimize technical heterogeneity. Although preprocessed data is often available in public repositories, it is best to start from the raw data and process all data sets using a unified pipeline. Conesa et al. (2016) provide an extensive review of the major steps involved in processing RNA-seq data.

Standard quality control, read mapping, and quantification procedures should be performed on raw RNA-Seq data at the start of any meta-analysis (Conesa et al., 2016). Briefly, raw reads should be analyzed for sequence quality, GC content, adaptor presence, overrepresented reads, and duplicated reads. Acceptable levels of duplicated content differ by organism but should be homogeneous for samples in the same experiment. It is recommended that outliers with more than 30% disagreement be discarded (Conesa et al., 2016). After initial quality checking of raw sequence reads, reads should be trimmed to remove adaptor sequences, low-quality reads, and poor-quality bases. Trimmed reads should then be mapped to the reference genome, and libraries with low read mapping percentage (organism dependent) removed from downstream analysis. After read mapping, quantification of transcript/gene expression should be performed.

One important, often overlooked, aspect of data preprocessing for meta-analysis is how to handle data generated using different RNA library preparation protocols. During RNA-Seq library preparation, the highly abundant ribosomal RNA (rRNA), which constitute over 90% of the RNA in the cell, are removed. The two most utilized methods for rRNA removal are polyA-selection and rRNA-depletion, which generate distinct fractions of the transcriptome (Sultan et al., 2014; Bush et al., 2017). Using two ovine RNA-Seq data sets, identical except for RNA selection method, Bush et al. (2017) demonstrate that although expression levels estimated by the two methods were correlated, rRNA depleted libraries systematically produced lower estimates of the relative expression of protein-coding genes. Using a common processing pipeline, in particular a common threshold for filtering lowly expressed genes, for data sets produced using differing RNA selection methods would then result in incompatible downstream data. However, equivalent expression levels between polyA-selected and rRNA-depleted libraries can be achieved using a combination of reference transcriptome filtering and a ratio-based correction.

### 3.3 Choosing a meta-analysis procedure

Meta-analysis procedures for gene expression data have been utilized since the early 2000s, with the earliest methods being proposed in the context of microarrays (Tseng et al., 2012). For microarray data, proposed meta-analysis methods include *p*-value combination (Marot et al., 2009), combining effect sizes (Choi et al., 2003), and ranking genes within each study (Breitling et al., 2004). Several reviews and comparisons of these methods are available (Hu et al., 2006; Hong and Breitling, 2008; Tseng et al., 2012).

Many of the meta-analysis techniques used for microarray data are not suited for RNA-Seq due to differences in the underlying structure of the data (Rau et al., 2014). Microarray data analyses utilize standard or moderated t-tests, which assume that data are continuous and can be approximated by Gaussian distributions after log-transformation (Smyth, 2004; Jaffrézic et al., 2007). RNA-Seq data, which come in the form of gene read counts, are often modeled by overdispersed Poisson (Auer and Doerge, 2011) or negative binomial distributions (Anders and Huber, 2010; Hardcastle and Kelley, 2010; Robinson et al., 2010). Under these models, calculation and interpretation of effect sizes is not straightforward (Rau et al., 2014).

**FIGURE 3:**
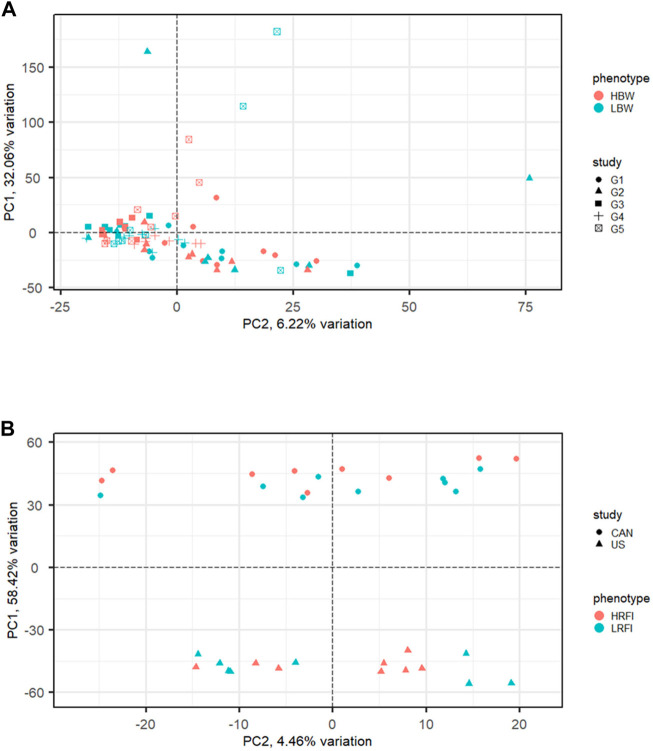
Examples of RNA-Seq data with low and high inter-study variability exhibited via principal components analysis (PCA). **(A)** Low inter-study variability in the muscle transcriptome of high (HBW) and low (LBW) body weight gain from five cohorts of steers reared at the U.S. Meat Animal Research Center (Keel et al., 2018). **(B)** High inter-study variability in the rumen transcriptome of cattle with high (HRFI) and low (LRFI) residual feed intake from a Canadian population and a United States population (Lindholm-Perry et al., 2022).

Commonly used meta-analysis procedures for RNA-Seq data include *p*-value combination and generalized linear models (GLM) with a fixed study effect. Performance of these methods has been linked to inter-study variability and the number of studies included in the analysis. Figure 3 illustrates examples of meta-analysis with low (Figure 3A) and high (Figure 3B) inter-study variability. Using both real data from human melanoma cells and simulated data, Rau et al. (2014) evaluated the performance of these methods for differing levels of inter-study variability, number of studies, and number of biological replicates per study. The methods considered included per-study *p*-value combination using the inverse normal (Liptak, 1958) and Fisher (1932) methods and a negative binomial GLM with fixed study effect. Results from the two *p*-value combination techniques were nearly identical. For low inter-study variability, the results from *p*-value combination were very close to those of the global GLM with study effect. As the inter-study variability increased, however, the gains in performance for *p*-value combination were significant compared to the global GLM, particularly for the analysis of data from more than two studies. Given these results, the use of *p*-value combination is likely the best choice for most meta-analyses. In some cases, it may be useful to utilize the union of results from the *p*-value and global GLM methods. In their meta-analysis of human melanoma samples, Rau et al. (2014) found that the sets of genes uniquely identified by Fisher *p*-value combination and global GLM, as well as the set of genes found in common, all appeared to include genes related to cancer or melanoma.

Additional data filtering is required if using the *p*-value combination approach. A critical underlying assumption for the statistics in *p*-value combination is that *p*-values for all genes in the per-study differential analyses are uniformly distributed under the null hypothesis (Rau et al., 2014). This assumption is, however, not always satisfied for RNA-Seq data; in particular, a peak is often observed for *p*-values close to one due to the discretization of *p*-values for very low counts (Rau et al., 2013). To circumvent this issue, weakly expressed genes should be filtered from the analysis. Data-based methods, such as HTSFilter (Rau et al., 2013), are preferred over ad-hoc filtering procedures, as they can account for differences between studies resulting from sequencing depth, intra-condition variability, and other technical factors.

## 4 Interpreting meta-analysis results

In this section, we discuss key considerations for interpreting the results of RNA-Seq meta-analysis.

### 4.1 Fold changes

It is not uncommon for genes in a meta-analysis to exhibit conflicting expression patterns among studies. For example, Lindholm-Perry et al. (2022) identified nearly 50% of rumen epithelial DEG (37 of 83 DEG) as discordant in their direction of expression between two unrelated, geographically distant populations of cattle. A similar phenomenon was observed in meta-analyses of muscle and mesenteric fat tissues across five cohorts of related cattle that were reared in a common facility (Keel et al., 2018; Lindholm-Perry et al., 2020).

Fold changes must be handled differently for global GLM and *p*-value combination techniques. For global GLM with study effect, fold changes are readily calculated using software such as DESeq2 (Love et al., 2014) and edgeR (Robinson et al., 2010). These fold changes have the same interpretation as those in single study analyses, as they are computed by averaging gene expression values across the conditions being compared. On the other hand, fold changes for the *p*-value combination are calculated on a per-study basis and can exhibit differing patterns between studies.

In the context of microarrays, approaches for overcoming conflicting expression patterns between studies for the inverse normal (Marot et al., 2009) and Fisher (Owen, 2009) *p*-value combination methods have been proposed. Both methods rely on the use of a two-tailed gene expression distribution, where under- and over-expressed genes reside in the tails of the distribution. However, for RNA-Seq data, where the data follows an overdispersed Poisson or negative binomial distribution, under- and over-expressed genes cannot be separated into distribution tails.

It has been suggested that DEG with differing expression patterns between studies be removed post hoc (Rau et al., 2014). However, genes with both concordant and discordant gene expression patterns across studies could be of interest. For example, sample collection time points will often be different between studies. The expression of some genes may be higher at earlier sampling time points and lower at later sampling time points. Hence, these genes would still be of biological interest and should be considered. Recently, there have been methodologies proposed for identifying and exploring discordant DEG sets (Lai et al., 2016; Ye et al., 2021), but additional research is needed to improve our understanding of underlying causes of these differences and how they contribute to the mechanisms of complex phenotypes.

### 4.2 Robustness of differentially expressed genes in *p*-value combination

Differential expression results, especially those derived from small sample sizes, are known to be susceptible to heterogeneity (Cui et al., 2021). As a result, reproducibility of DEG is often poor from study to study. For this reason, robustness of DEG arising from meta-analysis should be measured by replication validity rather than in independent data, such as cross-validation (Keel et al., 2018). Jackknife sensitivity analysis has been employed to measure the robustness of meta-analysis DEG (Ch’ng et al., 2015; Keel et al., 2018; Lindholm-Perry et al., 2020; Lindholm-Perry et al., 2022).

Jackknife sensitivity analysis consists of repeating the meta-analysis procedure multiple times, each time removing a single study from the analysis (Miller, 1974). Suppose a meta-analysis consists of N studies. A gene is said to pass a jackknife sensitivity test if it is identified as a DEG in the jackknife analysis. Genes that pass all N jackknife analyses can be considered highly robust, as their statistical significance is spread across studies, i.e., they are not being driven by any one study. Genes failing multiple jackknife studies can also be interpreted as robust, with a higher number of failed jackknife tests corresponding to greater robustness. The interpretation is derived as follows. A gene that fails only one jackknife analysis indicates that the meta *p*-value was being driven by the *p*-value of a single study, i.e., there is a significant amount of study bias. Similarly, genes that fail multiple jackknife analyses are being driven by *p*-values from multiple studies. This indicates a reduced level of study bias, i.e. a more robust result.

### 4.3 Downstream analysis

In RNA-Seq studies, DEG lists are subjected to several different downstream analyses with the intention of identifying gene signatures that link to the phenotype of interest. The most popular type of downstream analysis is gene set enrichment analysis (GSEA). In GSEA, the list of DEG is compared to a background gene set with known biological processes, such as gene ontology (GO) or biological pathways. An enrichment score, which indicates the degree by which a gene set is overrepresented in the list of DEG, is used to identify biological processes potentially associated with the phenotype (Subramanian et al., 2005).

There are three primary approaches to GSEA, overrepresentation analysis, functional class scoring, and pathway topology-based methods (Khatri et al., 2012). Over-representation analysis (ORA) approaches statistically evaluate the fraction of genes in a particular ontology/pathway found among the set of DEG. The most commonly used tests in ORA are based on the hypergeometric, chi-square, or binomial distribution (Huang et al., 2009). ORA methodologies only utilize lists of DEG, disregarding other quantitative measures of the genes such as fold change. In functional class scoring (FCS) methods, use a similar approach to ORA but adds in quantitative information from the genes (Mootha et al., 2003). A gene score is calculated for each gene, and individual gene scores are used to calculate a gene set score. Significance of gene set scores is assessed and differentially enriched gene sets are reported. Topology-based (TB) methods for GSEA go a step further by utilizing the network structure of a gene pathway to quantify a gene’s importance to a given pathway (Draghici et al., 2007).

Currently, researchers are using the same downstream analyses for DEG sets coming from meta-analysis as are used for single studies. While ORA tools are directly applicable to meta-analysis results, current FCS and TB methodologies are not since they are designed to be used with a single set of information for each gene, e.g., a single fold change. Integration of DEG expression profiles across studies in a meta-analysis could shed light on the molecular mechanisms governing phenotypes across environments, especially for DEG exhibiting discordant fold changes across studies. To our knowledge, there are currently no bioinformatic approaches that integrate meta-analysis results in this way. Future development of algorithms to incorporate this type of data would help harness the full potential of gene expression meta-analyses.

## 5 Additional discussion points

### 5.1 Other applications of RNA-Seq meta-analysis in livestock

As mentioned in the introduction, the term meta-analysis is used to encompass many different types of analyses, all with an underlying common goal of synthesizing results across different empirical studies. In this review, we focused specifically on meta-analysis procedures for analyzing differential gene expression. In addition to this approach, meta-analysis procedures have been used to integrate analyses of functional genome information with large-scale GWAS data to discover trait- and disease-relevant tissues and cell types.

In an effort to establish connections at the RNA level between tissue/cell types and complex traits, Fang et al. (2020) uniformly assembled and analyzed, via meta-analysis techniques, over 700 bovine RNA-Seq data sets, encompassing 91 tissues and cell types. Tissue- and cell-specific genes were detected and classified in terms of their biological characteristics such as biological function, DNA methylation, and evolution. Tissue-specific genes were integrated with large scale GWAS data to detect candidate genes for 45 complex, economically relevant traits via transcriptome-wide analysis study (TWAS). This study laid the groundwork for the FarmGTEx project, which seeks to characterize tissue-specific gene expression and genetic regulation in livestock.

In the seminal manuscript from the FarmGTEx project, Liu et al. (2021) built the Cattle GTEx (http://cgtex.roslin.ed.ac.uk/) utilizing 11,642 bovine RNA-Seq data sets. Transcriptome landscape across over 100 tissues was described, and gene expression in different tissues was linked to 43 economically relevant traits via TWAS and colocalization analyses. Similar efforts in swine (PigGTEx) are ongoing in the FarmGTEx project (Fang, personal communication, 24 May 2022). With more transcriptomics data becoming available across diverse tissues in livestock in the near future, from projects such as FarmGTEx and FAANG (Giuffra et al., 2019), the use of this type of meta-analysis will be powerful in providing novel insights into the genetic and biological mechanisms underpinning traits and thus enhancing genomic improvement programs.

In addition to its use in integrating multiple RNA-Seq experiments with GWAS data, network meta-analysis has been utilized for differential expression analysis across independent livestock studies. Fonseca et al. (2020) identified DEG between high- and sub-fertile cows in two independent studies and utilized network meta-analysis (Winter et al., 2019) to obtain combined test-statistics for each of the genes. These test-statistics are like those described in Section 3.3. The use of network meta-analysis for RNA-Seq data sets is relatively new and, to our knowledge, has not been systematically compared to the meta-analysis procedures described in Section 3.

### 5.2 New RNA-Seq protocols to reduce costs in large-scale expression studies

There are many different options for library preparation and sequencing that users must consider when designing an RNA-Seq experiment. One of the most critical and costly steps in RNA-Seq is the construction of the cDNA library (Hou et al., 2015). In the classic whole-transcriptome approach, cDNA are generated from reverse transcribing randomly sheared fragments of the extracted mRNA. Although these approaches are generally considered unbiased, there are some subtle biases that are introduced, such as differentially expressed genes being more likely to be enriched for longer transcripts (Oshlack & Wakefield, 2009). Recently, 3’ RNA-Seq have been introduced to address this bias. In addition, 3’ RNA-Seq methods, such as QuantSeq (Moll et al., 2014), have been shown to significantly reduce associated financial and labor costs associated with library preparation. These cost reductions will make large-scale expression studies more feasible, especially in livestock where budget is typically the most limiting factor.

The most widely deployed 3’ RNA-Seq method, QuantSeq, produces fragments for sequencing close to the 3’ end of polyadenated [poly(A)] mRNA, generally from the last exon or the 3’ untranslated region (UTR) (Moll et al., 2014). Total RNA is used as input with no prior poly(A) enrichment or rRNA depletion. QuantSeq differs from traditional RNA-Seq in that it sequences a smaller part of the transcript and produces only one fragment per transcript. Therefore, less sequencing is required. In fact, the QuantSeq vendor, Lexogen, recommends sequencing only 10 million reads per sample for mammalian transcriptomics (Corley et al., 2019). It has been shown that gene expression levels in QuantSeq and Illumina TruSeq are strongly correlated (Corley et al., 2019). The major limitation of QuantSeq is that it is restricted to assessing gene expression changes of poly(A) mRNA. It does not provide information regarding mutations or novel transcripts. For longer transcripts with many isoforms or non-poly(A) transcripts, traditional RNA-Seq may be more appropriate. To date, QuantSeq has been deployed in transcriptomic studies of multiple livestock species, including cattle (Pascottini et al., 2021; Beak and Baik, 2022; Busato et al., 2022; Pedroza et al., 2022), sheep (Kubik et al., 2018), chicken (Ibrahim et al., 2021) and pigs (Dong et al., 2018; Kroeske et al., 2021; Schroyen et al., 2021; Kramer et al., 2022).

One drawback to QuantSeq, as well as traditional RNA-Seq library preparation, is that the user needs to process each sample on an individual basis. To address this limitation, early multiplexing protocols, which label individual samples during the reverse transcription reaction, have been developed (Alpern et al., 2019). Once individual samples have been labeled, they are pooled, and the remainder of the library processing steps are performed in bulk. This shortens the time and cost of library preparation. Early multiplexing methodologies are available for traditional RNA-Seq library preparation (BRB-seq; Alpern et al., 2019), as well as 3’ RNA-Seq library preparation (3’Pool-seq; Sholder et al., 2020). Utilization of these approaches results in a significant reduction in cost per library for library preparation and sequencing. In fact, 3’Pool-seq can reduce the per-library cost of library preparation and sequencing from over $160 in traditional RNA-Seq to under $15 (Sholder et al., 2020).

The technologies mentioned above have many strengths, including reducing cost and streamlining experimental procedures. They will facilitate new opportunities for future large-scale transcriptomics studies in livestock. Future work should focus on evaluating and comparing these methodologies in terms of various robustness metrics, including gene expression quantification accuracy and DEG detection. Understanding how data generated from these approaches compares with traditional RNA-Seq data will be crucial for conducting meta-analyses across studies with differing RNA-Seq technologies.

## 6 Conclusion

Although the cost of next-generation sequencing technologies continues to decrease, livestock transcriptomic studies are often performed on a small number of samples due to financial constraints. Small sample sizes result in decreased detection power for DEG. Utilization of meta-analysis procedures to combine and analyze data across multiple related studies can increase statistical power and robustness of results. The aim of this paper was to present and discuss several key considerations for meta-analysis of livestock RNA-Seq data.

The use of meta-analysis in livestock transcriptomic data should provide identification of DEG that underly complex phenotypes as they account for some of the potentially confounding issues that may influence gene expression in a single study such as, sire lines, environment, and management. Differentially expressed genes identified from analysis across multiple populations will likely be more robust biological markers. Additional research is needed to develop techniques for downstream analysis of meta-DEG that integrate expression profiles across studies.
